# Some Thoughts for Nurses

**Published:** 1919-11-22

**Authors:** 


### SOME THOUGHTS FOR NURSES.

### Seek Time for These Things.

Art, music, drama, travel, hobbies of every kind, the progress of the human race, and the aspirations of the inventor—all that interests the cultivated intelligent man or woman—how wonderful if nurses had time for these things! Miss Milne has eloquently opened up the possibilities of the local centre, and lucky, indeed, are the nurses who will have such opportunities as she outlines. But torn betwixt two sets of values, some of us find we are unable to throw ourselves heart and soul into the establishment of either.

### The Conventual Ideal.

One ideal practically demands that a woman who decides to be a nurse shall forsake the world of healthy men and women, shall give up all pursuits and interests outside the hospital walls, and be almost as completely cut off from normal life as if she had entered a convent. Beautiful, pure, attractive to some natures as this may be, is it really desirable? Possibly only in a training-school large enough to form a little world in itself, is it wise even here? Is it expedient from the point of view of the profession?

### Nurses as Human Beings.

Keenness, gentleness and strength, patience, hard work, accuracy and attention to detail, powers of observation, kindness and unselfishness, consideration, and understanding—all these we need as nurses, but we need them also as tennis-players or partakers in sport of any kind, as musicians or singers, actors or artists, needlewomen, literary students—in short, as human beings:

### Let Us Be.

Nurses devoted, patient, conscientious and wholehearted let us be, seeking not our own but another's good. Women let us also be, responsive, alert, intelligent, ever ready to assimilate new impressions and to see wider points of view, alive to the beauty and richness of the world around us and to the infinitely varied manifestations of life.

